# Algal Proteins: Extraction, Application, and Challenges Concerning Production

**DOI:** 10.3390/foods6050033

**Published:** 2017-04-26

**Authors:** Stephen Bleakley, Maria Hayes

**Affiliations:** 1 Food Biosciences Department, Teagasc Ashtown Food Research Centre, Ashtown, Dublin D15 KN3K, Ireland; Stephen.Bleakley@teagasc.ie; 2 School of Biological Sciences, College of Sciences and Health and Environment, Sustainability and Health Institute, Dublin Institute of Technology, Kevin Street, Dublin D08 NF82, Ireland

**Keywords:** seaweed, microalgae, peptides, phycobiliproteins, biorefinery, bioavailability, extraction methods, legislation

## Abstract

Population growth combined with increasingly limited resources of arable land and fresh water has resulted in a need for alternative protein sources. Macroalgae (seaweed) and microalgae are examples of under-exploited “crops”. Algae do not compete with traditional food crops for space and resources. This review details the characteristics of commonly consumed algae, as well as their potential for use as a protein source based on their protein quality, amino acid composition, and digestibility. Protein extraction methods applied to algae to date, including enzymatic hydrolysis, physical processes, and chemical extraction and novel methods such as ultrasound-assisted extraction, pulsed electric field, and microwave-assisted extraction are discussed. Moreover, existing protein enrichment methods used in the dairy industry and the potential of these methods to generate high value ingredients from algae, such as bioactive peptides and functional ingredients are discussed. Applications of algae in human nutrition, animal feed, and aquaculture are examined.

## 1. Introduction

The global population is expected to increase by over a third (2.3 billion people) by 2050, requiring an estimated 70% increase in food production [1]. A combination of improved agricultural food production methods and an increase of average per capita income have led to a decrease in global hunger over the last half-century, despite a doubling of the world’s population [1]. However, worldwide food production is now facing a greater challenge than ever before. Previously utilised methods of intensifying agriculture will soon no longer be an option due to the high impact trade-offs they have on the environment, including fragmenting natural habitats and threatening biodiversity, production of greenhouse gases from land clearing, fertilisers and animal livestock production, and nutrient run-off from fertiliser damaging marine, freshwater and terrestrial ecosystems [2]. In particular, protein is one of the main nutrients that will be in short supply in the future. Alternative protein sources and production methods are required to fulfil the demand of consumers and to meet predicted global protein requirements.

Seaweed and microalgae are considered a viable source of protein. Some species of seaweed and microalgae are known to contain protein levels similar to those of traditional protein sources, such as meat, egg, soybean, and milk [3,4]. Algae use for protein production has several benefits over traditional high-protein crop use in terms of productivity and nutritional value. Seaweed and microalgae have higher protein yield per unit area (2.5–7.5 tons/Ha/year and 4–15 tons/Ha/year, respectively) compared to terrestrial crops, such as soybean, pulse legumes, and wheat (0.6–1.2 tons/Ha/year, 1–2 tons/Ha/year, and 1.1 tons/Ha/year, respectively) [5]. Terrestrial agriculture already requires approximately 75% of the total global freshwater with animal protein in particular requiring 100 times more water than if the equivalent amount of protein was produced from plant sources [6,7]. Marine algae do not require freshwater or arable land to grow, maximising resources that can be used for additional food production or other cash crops [5]. Furthermore, due to their harsh environment and phototropic life, algae are often exposed to high oxidative and free-radical stresses [8]. This has led to the evolution of natural protective systems, such as the production of pigments (e.g., carotenes, chlorophylls, and phycobiliproteins) and polyphenols (e.g., catechins, flavonols, and phlorotannins), which can impart health benefits to the consumer when eaten [9,10].

However, widespread use of seaweed and microalgae is limited by a number of factors including; harvesting access and rights, seasonality and geographical location of algae, as well as the availability of scalable production methods for protein isolation from algae. Current processes of algal protein isolation are time-consuming and economically unviable [11]. The objective of this paper is therefore to discuss the value of algal proteins as a source of human nutrition, functional foods and animal feed, as well as describe current extraction methods and novel processing technologies that are used in dairy processing which may be employed to make algae a viable source of protein ingredients.

### 1.1. Characteristics of Seaweed

Algae are a diverse group of species which can be broadly described as oxygen-producing, photosynthetic, unicellular or multicellular organisms excluding embryophyte terrestrial plants and lichens [12]. Macroalgae can be divided into three main taxonomic groups based on their pigmentation; Phaeophyta (brown algae), Chlorophyta (green algae), and Rhodophyta (red algae) [13].

According the Food Balance sheets published by the Food and Agriculture Organisation of the United Nations (FAO), the Republic of Korea is the greatest consumer of seaweed (22.41 kg/capita/year in 2013), followed by China and Japan [14]. Production of farmed seaweed has more than doubled worldwide since 2000, with particular expansion seen in Indonesia due to their vast areas of shallow sunlight coasts suitable for culture sites [15]. The culture of Japanese kelp, *Laminaria japonica*, has traditionally been the most extensively farmed cold-water species. This was surpassed by the tropical *Eucheuma* seaweeds (*Kappaphycus alvarezii* and *Eucheuma* spp.) in 2010. The other most commonly farmed seaweed species are *Gracilaria* spp., *Undaria* sp., and *Porphyra* spp. [15].

Brown algae are distinguished by the presence of the pigment fucoxanthin, which is responsible for the distinctive olive-brown colour that lends this group its name [13]. Brown algae are also unique among algae as they are only found in multicellular form [16]. There are approximately 1500–2000 species of brown algae worldwide [17]. Some species, such as *Macrocystis pyrifera* (giant kelp), play an important role in the ecosystem, growing up to 20 m and forming underwater kelp forests [18]. There are also many other species that have been exploited for human consumption, including *Undaria pinnatifida* (wakame), *Hizikia fusiformis* (hijiki), and *Laminaria japonica* (kombu) [19]. Several types of brown algae are used for animal feed, including *Laminaria digitata* (oarweed), *Ascophyllum nodosum* (rockweed) and *Fucus vesiculosus* (bladder wrack) [3].

Green algae are a diverse group of approximately 8000 species, consisting of the divisions Chlorophyta and Charophyta [20]. Chlorophyta are a large group of unicellular and multicellular algae, while charophyta are a smaller group of exclusively freshwater, multicellular green algae from which it is believed that Embryophyta (terrestrial plants) evolved from [21]. Green algae obtain their pigmentation from chlorophyll a and b, as well as other pigments including β-carotene and xanthophylls [13]. The most commonly consumed species of green algae are *Ulva* spp. including *U. lactuca* (sea lettuce), *U. intestinalis*, and *U. compressa*.

Red algae are a large group of mostly multicellular macroalgae with approximately 6000 species [13]. Red algae are characterised by the presence of phycobilins, which are responsible for their red colour [13]. Species of Irish moss, such as *Chondrus crispus* and *Mastocarpus stellatus*, are exploited for their production of carrageenan [22]. Other species of red algae, including *Porphyra tenera* (nori) and *Palmaria palmata* (dulse), are among the highest consumed species of seaweed in Asia, as well as Western countries, due to their high protein content and their delectable flavour [23]. In particular, *Porphyra tenera* is used in the production of sushi.

### 1.2. Characteristics of Microalgae

Microalgae are unicellular, microscopic organisms that are also considered as a viable alternative protein source. The most abundant microalgal divisions are Bacillariophyta (diatoms), Chlorophyta (green algae), Chrysophyta (golden algae), and Cyanophyta (blue-green algae). Microalgae are a hugely diverse group containing approximately 200,000 species [24]. Several of these species are currently exploited for a variety of biotechnological purposes, including cosmeceuticals, animal feed, fatty acids, alginates, wastewater treatment, and biofuel [9,25,26]. Furthermore, *Arthrospira platensis* (*Spirulina*), and *Chlorella vulgaris* (*Chlorella*) are also sold as functional foods due to their high vitamin and mineral content and as they are generally regarded as safe (GRAS) by the European Food Safety Authority (EFSA) [25]. Despite the relatively low quantity of microalgae produced annually compared to that of seaweed (5000 tonnes dry matter per year versus 7.5 × 10^6^ tonnes dry matter per year, respectively), whole microalgal biomass and added-value compounds are economically valuable, representing a global turnover of about US $1.25 × 10^9^ per year, compared to annual seaweed turnover of US $6 × 10^9^ [9].

*Arthrospira platensis* is a filamentous Cyanobacterium that has among the highest recorded protein content of any whole food [27]. *Arthrospira* sp. was originally referred to as *Spirulina* sp. until a re-examination showed that they are actually a distinct genus [9]. However, due to its widely publicised use as a food and dietary supplement, the term *Spirulina* is often used interchangeably with *Arthrospira*.

*Chlorella* spp. are spherical members of the phylum Chlorophyta (green algae) that have also seen increased popularity as a food supplement in recent times. *Chlorella vulgaris* is the most commonly exploited industry species due to its high protein content (51%–58% dry weight; dw) and favourable essential amino acid composition [28]. *Chlorella* also contains many other beneficial nutrients, including β-1,3-glucan, vitamins (B-complex and ascorbic acid), minerals (potassium, sodium, magnesium, iron, and calcium), β-carotene, chlorophyll, and *Chlorella* growth factor (CGF) [29].

## 2. Protein Quality

### 2.1. Amino Acid Composition

The quality of proteins can vary dramatically, depending on digestibility and the availability of essential amino acids [30]. Animal sources of protein are generally considered as complete proteins, as they are a rich source of essential amino acids (EAAs) which the human body is unable to biosynthesise. Alternatively, plant proteins are often considered an incomplete protein source as they commonly lack one or more of the essential amino acids, including histidine, isoleucine, leucine, lysine, methionine, phenylalanine, threonine, tryptophan, and valine [31]. However, the lacking essential amino acid(s) in plant-based proteins can differ, meaning that an individual should be able to obtain a sufficient quantity of all essential amino acids if they consume a varied diet of plant proteins from fruit, vegetables, grains, and legumes [32]. Plant-based proteins are also typically harder to digest than animal proteins, due to their high concentration of insoluble polysaccharides. Despite this, there are increasing concerns about the high levels of saturated fats and cholesterol found in foods of animal origin, which are linked to the development of cardiovascular disease and diabetes. This has led to nutritionists and organisations, such as the FAO, recommending a more varied diet rich in plant-based proteins [33].

Algae are generally regarded as a viable protein source, with EAA composition meeting FAO requirements and they are often on par with other protein sources, such as soybean and egg [3,33]. The lack of widespread consumption of marine algae has led to a shortage of in vivo research regarding the ileal digestion of algae, thus limiting the comparison of algal protein quality between different algae species, as well as with other protein sources [34]. Nevertheless, tryptophan and lysine are often limiting amino acids in most algae species [35,36,37]. Furthermore, leucine and isoleucine are commonly found in low concentrations in red species of algae, while methionine, cysteine, and lysine are often limiting in brown algae species [35,38]. Cysteine typically occurs at low levels in many seaweed species, and is often not detectable [39]. Aspartic acid and glutamic acid constitute a relatively large proportion of the total amino acids in many seaweed species, largely contributing to the distinctive ‘umami’ taste associated with seaweed [40]. For example, these two amino acids have been reported to represent 22%–44% of total amino acids in *Fucus* sp. and 26%–32% in *Ulva* sp. [3].

### 2.2. Algal Protein Digestibility

**Figure 1:**
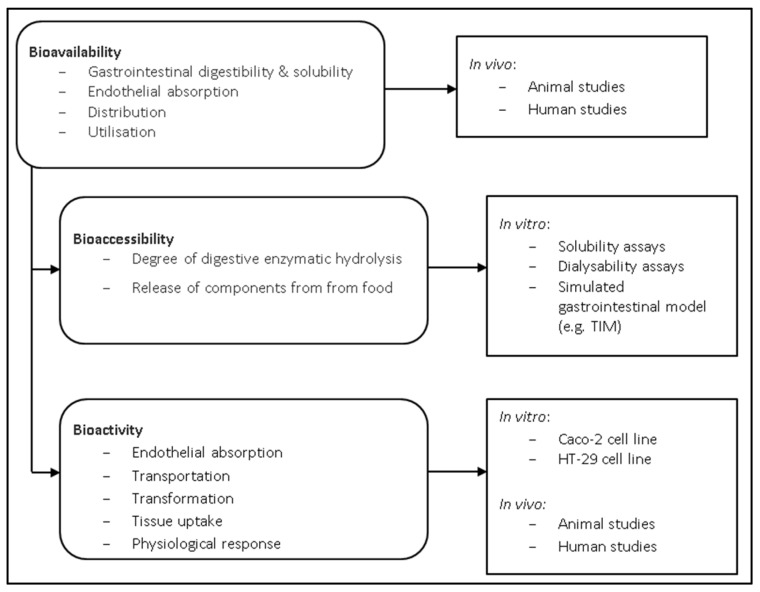
Schematic representation of digestion and methods that may be used to determine bioavailability, bio-accessibility and bioactivity of proteins and other foods. Adapted from Carbonell-Capella et al. [47]. TIM: TNOASR’s intestinal model; TNOASR: The Netherlands Organization for Applied Scientific Research.

Bioavailability can be described as the fraction of ingested food components that is available at the target site of action for utilisation in various physiological functions [41]. Bioavailability entails the entire process following food element consumption, including digestibility and solubility of the food element in the gastrointestinal tract, absorption/assimilation of the food element across the intestinal epithelial cells and into the circulatory system, and finally, incorporation into the target site of utilisation (Figure 1) [42]. Studies examining the bioavailability of food elements are therefore required to incorporate in vivo experiments. One in vivo study evaluated the bioavailability of *P. tenera* and *U. pinnatifida* in Wistar rats, which reported that the fibre in seaweed had a negative impact on protein intake digestibility and food efficiency [43]. Similarly, *L. japonica* was also reported to decrease protein digestibility in rats, although interestingly, digestibility ended up being comparable with the control diet after 3 weeks as the rats appeared to adapt to the high fibre diet [44]. It is thought that phlorotannins and high polysaccharide content are the main factors which negatively impact the digestibility of algal proteins [45,46].

Bioavailability can be further divided into two different stages; bioaccessibility and bioactivity. Bioaccessibility involves examining the fraction of particular components that are released from the whole food matrix within the gastrointestinal tract in order to identify elements that are accessible for further absorption [48]. Bioactivity refers to the assimilation of a food element across intestinal cells, transport of the element to the target site, interaction of the element with the target site, any necessary biotransformation of the food element, and the physiological response created as a result of incorporation of the element with the target site (Figure 1) [49]. There are many factors affecting digestibility which can make in vitro studies difficult, including the macronutrient composition, enzyme specificity, anti-nutritional factors, fibre, and varying absorptive capacities at different stages within the gastrointestinal tract [30]. Nevertheless, in vitro studies serve as a useful preliminary screening tool to identify promising food matrices, growing conditions, and processing methods [48,50,51].

Methods for assessing in vitro digestibility are typically divided into four categories, including solubility, dialysability, gastrointestinal chambers, and cell models [48]. Only small, soluble molecules can be absorbed in the small intestine, which can be evaluated by methods such as atomic absorption spectrophotometry, mass spectrometry, or high-performance liquid chromatography [48]. Dialysability is a direct measure of a food components ability to cross a membrane, although dialysability assays have typically been carried out on micronutrients, including iron, zinc, magnesium and calcium [52].

Bioaccessibility in vitro studies are typically accomplished using either static or dynamic measuring systems. Static systems are the more basic of the two, measuring the release of free amino acids from dietary proteins following hydrolysis from gastrointestinal enzymes under discrete pH and temperature [49]. Static systems have the advantage of being easily implemented, low cost, and high throughput, but have the disadvantage of being unrealistic for normal gastrointestinal physiological processes. Alternatively, dynamic systems use computer systems to tightly regulate pH, temperature, enzyme addition, mixing and residence times within chambers in order to more closely mimic gastrointestinal digestion [48]. These systems model gastric physiology more accurately, but have the drawbacks of being costly and low throughput, limiting their routine use.

The Netherlands Organization for Applied Scientific Research (TNOASR) has developed two similar dynamic gastrointestinal models, called TNOASR’s intestinal model (TIM-1 and TIM-2) [48]. The TIM-1 system contains several compartments used to mimic the effect of the stomach and small intestine (including duodenum, jejunum, and ileum) [53]. TIM-2 focuses on the large intestine, serving as a tool to study the effect of microbial fermentation and nutrient absorption in the colon [54]. In both models, aliquots can be taken from any chamber at any given time [48]. Additional in vitro gastrointestinal models have also been described to study digestion and microbiota colonisation [55,56,57,58]. However, one such issue that plagues digestibility studies is the lack of consistency occurring in differing methodologies, making the resulting data difficult to compare [59]. INFOGEST is an action granted by the European Cooperation in Science and Technology (COST) and was developed to help overcome this hurdle. INFOGEST is a static in vitro digestion model which aims to harmonise the methods used to assess digestibility, allowing for better comparisons between studies [60,61].

Various in vitro cell culture methods have been utilised to simulate a food component’s ability to be assimilated within the intestine, the first component within bioactivity (Figure 1). Caco-2 cells, a cell line derived from human colonic adenocarcinoma, are by far the most commonly used [52,62]. HT-29 is another human colon carcinoma cell line that has been used to study epithelial transport, although it is rarely used [63]. The co-culture of Caco-2 cells with a human mucous-producing cell line, such as HT29-MTX, has been suggested to more closely resemble in vivo conditions [64].

Similar to the previously described in vivo protein quality studies [43,44], in vitro bioaccessibility studies also appear to suggest that unprocessed seaweed proteins have reduced digestibility compared to that of other protein sources. For example, the seaweed species *P. tenera*, *U. pinnatifida*, and *Ulva pertusa* have reported in vitro bioaccessibility of 78%, 87%, and 95%, respectively, expressed as a percentage of casein bioaccessibility (100%) [3]. *U. lactuca* has been shown to have an in vitro digestibility of 85.7% ± 1.9%, while the red seaweeds *Hypnea charoides* and *H. japonica* have high digestibility of 88.7% ± 0.7% and 88.9% ± 1.4%, respectively [65]. Comparable results for *U. lactuca* were found with in vitro simulated ileal digestibility of 82.3% [66]. Tibbetts and colleagues (2016) reported significantly greater in vitro digestibility in red seaweeds (83%–87%) compared to brown seaweeds (78.7%–82%) [67]. These results demonstrate that seaweed proteins have comparable in vitro digestibility compared to that of other commonly consumed plants, including grains (69%–84%), legumes (72%–92%), fruits (72%–92%), and vegetables (68%–80%) [67].

The digestibility of microalgae is poorly examined within the literature, including in vitro bioaccessibility studies. However, microalgae appear to have similar digestibility to that of seaweed, with *Scenedesmus obliquus*, *Spirulina* sp., *Chlorella* sp. having digestibility coefficient values of 88.0%, 77.6%, and 76.6%, respectively [28]. This is in comparison to protein sources such as casein and egg with a digestibility coefficient of 95.1% and 94.2%.

## 3. Protein Extraction Methods

### 3.1. Conventional Protein Extraction Methods

Seaweed and microalgae have poor protein digestibility in their raw, unprocessed form and it is for this reason that great emphasis has been placed on developing improved methods for algal protein extraction in order to improve their bioavailability. Algal proteins and their extraction is a relatively poorly studied topic compared to proteins from other crops [68]. Algal proteins are conventionally extracted by means of aqueous, acidic, and alkaline methods, followed by several rounds of centrifugation and recovery using techniques such as ultrafiltration, precipitation, or chromatography [69]. Chemical extraction methods, such as two-phase acid and alkali treatments, have been especially efficient for extracting proteins from *A. nodosum*, *Ulva* spp. and *L. digitata* (Table 1) [69,70,71].

However, the successful extraction of algal proteins can be greatly influenced by the availability of the protein molecules, which can be substantially hindered by high viscosity and anionic cell-wall polysaccharides, such as alginates in brown seaweed and carrageenans in red seaweed [72]. Cell disruption methods and the inclusion of selected chemical reagents are therefore used in order to improve the efficiency of algal protein extraction. Some examples of conventional methods that are commonly utilised include mechanical grinding, osmotic shock, ultrasonic treatment, and polysaccharidases-aided hydrolysis (Table 1) [73].

#### 3.1.1. Physical Processes

Barbarino and Lourenço (2005) reported that physical grinding with the use of a Potter homogeniser significantly increased protein extraction yield from *Porphyra acanthophora* var. *acanthophora*, *Sargassum vulgare*, and *Ulva fasciata* following immersion in ultra-pure water (Table 1) [68]. Alternatively, osmotic stress has also been reported to improve extraction of algal proteins efficiency [45,75]. Osmotic shock was reported to yield a significantly higher concentration of water soluble proteins from *P. palmata* (1.02 ± 0.07 g/100 g) compared to high shear force with an Ultra-turrax^®^ T25 Basic tool (IKA^®^, Staufen, Germany) (0.74 ± 0.02 g/100 g) [73]. However, there was no significant difference in the amount of total protein extracted between the two methods (6.77 versus 6.92 g/100 g). Alternatively, the use of polysaccharidases was reported to be a more promising method of protein extraction, with a concentration of 11.57 ± 0.08 g/100 g *P. palmata*, equating to a yield of 67% (Table 1) [73].

#### 3.1.2. Enzymatic Hydrolysis

Seaweed is rich in several types of polysaccharides, including cellulose, galactans, xylans, fucoidan, laminarin, alginates, carrageenans, and floridean starch [22]. These polysaccharides can reduce the availability of algal proteins and decrease protein extraction efficiency [68]. Enzymes such as polysaccharidases can therefore be applied as a cell disruption treatment prior to protein extraction in order to increase protein yield (Table 1). Several polysaccharidases (κ-carrageenase, β-agarase, xylanase, cellulase) were used in protein extractions from the red seaweed species *C. crispus*, *Gracilaria verrucosa*, and *P. palmata* as a method of combating the tough cell wall [74]. Hydrolysis of *C. crispus* with carrageenase and cellulase increased protein yield ten-fold compared to the enzyme-free procedure, while the highest yield from *P. palmata* was obtained with xylanase. Similarly, hydrolysis of *P. palmata* with xylanase and cellulase was demonstrated to yield a ten-fold increase in the phycoerythrin pigment protein compared to mechanical extraction [46]. Harnedy and Fitzgerald (2013) also increased protein yield from *P. palmata* with xylanase, although they reported that the high enzyme:substrate concentration required (48.0 × 10^3^ units/100 g) may not be commercially feasible at an industrial scale. Combining multiple extraction methods may also help to improve algal protein extraction. Combining enzymatic hydrolysis with alkaline extraction increased protein yield 1.63-fold compared to alkaline extraction alone in *P. palmata* [76].

### 3.2. Current Protein Extraction Methods

Protein extraction methods used on algae to date are limited for commercial use due to concerns with up-scaling. Conventional mechanical and enzymatic methods for protein extraction may also affect the integrity of extracted algal proteins due to the release of proteases from cytosolic vacuoles [77]. Furthermore, these methods are also laborious and time consuming [69]. Improved extraction methods of cell disruption and extraction are therefore required. Pre-treatment with cell-disruption techniques aid the breakdown of the tough algal cell wall, increasing the availability of proteins and other high-value components for later protein extraction. Some examples of novel protein extraction methods include ultrasound-assisted extraction, pulsed electric field, and microwave-assisted extraction [13].

#### 3.2.1. Ultrasound-Assisted Extraction

Ultrasound-assisted extraction (UAE) can be applied to food sources for a number of applications, including modification of plant micronutrients to improve bioavailability, simultaneous extraction and encapsulation, quenching radical sonochemistry to avoid degradation of bioactives, and increasing bioactivity of phenolics and carotenoids by targeted hydroxylation [78]. The degradative effect of radical sonochemistry, which is the most relevant aspect in terms of improving bioavailability of algal proteins, is not produced by the ultrasound waves, but rather by the formation, growth, and implosion of bubbles formed by what is known as acoustic cavitation [79]. The violent implosion of these bubbles creates microscopic regions of extreme pressure and temperature, resulting in the chemical excitation of the sonicated liquid and its contents, facilitating the particle breakdown and degradation of the target compound [80]. The major advantages of UAE are its fast processing time, non-thermal properties, and low solvent consumption, resulting in a higher purity final product with reduced downstream processing required [81].

Ultrasound pre-treatment was reported to increase protein extraction of *Ascophyllum nodosum* with acid and alkaline treatment alone by 540% and 27%, respectively, as well as reduce processing time from 60 min to 10 min [69]. This dramatic increase in protein yield was suggested to be due to acid hydrolysis alone being insufficient to erode the tough cell wall. Ultrasound-aided extraction was also evaluated in microalgae for a number of value-added components, although there have been relatively few studies that have focused on ultrasound for improved protein extraction [82,83]. Keris-Sen and colleagues (2014) reported that ultrasound at a power intensity of 0.4 kWh∙L^−1^ yielded the optimum concentrations of proteins from wastewater treatment microalgae from the Chlorococcales order of the Chlorophyceae class (e.g., *Scenedesmus* sp.) [84]. Ultrasound treatment of *C. vulgaris* significantly increased crude protein digestibility in rats compared to electroporated and untreated spray-dried *C. vulgaris* (56.7% ± 13.7%, 44.3% ± 7.5%, and 46.9% ± 12.7%, respectively), as well as significantly improving protein efficiency ratio and nitrogen balance [85]. Additionally, there were no adverse effects to the histology of major organs upon prolonged consumption of ultrasound-treated microalgae, and thus it has been suggested as a viable pre-treatment method for the food industry [86]. Alternating two counter-current frequencies has also been suggested as a viable method for further improving protein extraction, as demonstrated by a 50% increase in yield and 18% reduction in extraction time using 15 and 20 kHz in *Porphyra yezoensis* compared to mono-frequency ultrasound-assisted extraction [87].

#### 3.2.2. Pulsed Electric Field

Pulsed electric field (PEF) has been used as a cell disruption technique in microalgae, although its primary use has thus far been for the extraction of lipids for conversion to biofuel [88]. PEF involves applying high electric currents in order to perforate a cell wall or cell membrane, causing reversible or irreversible electroporation [88]. Electroporation enables the introduction of various foreign components to cells, including DNA, proteins, and drugs [89]. PEF is a fast and green technology for inactivating microorganisms by irreversible electroporation and aiding the release of intracellular contents of plant cells [90,91]. However, conductivity and electrode gap are factors that could possibly limit this technology for up-scaling [92].

Goettel and colleagues (2013) were among the first to report the use of PEF as a means of extracting multiple intracellular components from algae [93]. Since then, PEF has been demonstrated to increase the yield of several high-value microalgae components, including lipids, carbohydrates, carotenoids and chlorophyll [94,95,96,97,98]. Protein yield from *Chlorella* sp. and *Spirulina* sp. was reported to increase by 27% and 13%, respectively, following PEF-treatment at 15 kV/cm and 100 kJ/kg [99]. Coustets and colleagues (2013) also reported significantly increased protein extraction in *C. vulgaris* and *Nannochloropsis salina* following PEF-assisted extraction, allowing for the extraction of intact cytosolic proteins [100].

#### 3.2.3. Other

Microwave-assisted extraction (MAE) involves heating a material, causing moisture to evaporate, thus creating bubbles under high pressure which can then rupture to disrupt cell contents [82]. Increased levels of soluble proteins were extracted from a microalgae biomass containing green microalgae (*Stigeoclonium* sp. and *Monoraphidium* sp.) and diatoms (*Nitzschia* sp. and *Navicula* sp.) using microwave pre-treatment compared to ultrasound [101]. MAE has attracted attention for extraction of compounds due low energy efficiency, although its use in algae may be limited by impaired function with dried samples [81].

Alternatively, sub- and supercritical fluid extraction techniques have gained popularity in recent decades as extraction methods. Subcritical water extraction (SWE) involves using hot water (100–374 °C) under high pressure (~10 bar) to maintain water in a liquid state [102]. Alternatively, supercritical fluid extraction (SFE) is a technique that heats a fluid above its critical point, making it supercritical. Under supercritical conditions, the properties of the fluid become indistinguishable from its gaseous state, with a density similar to a fluid, but viscosity matching that of gas [102]. SFE typically utilises carbon dioxide (CO_2_), making it a relatively ‘green’ technology with low solvent consumption [81]. However, SWE and SFE both require high investment costs for equipment and have typically only been used in algae to date for the extraction of lipids [102].

### 3.3. Enrichment Methods—Membrane Filtration

Membrane technologies are widely used in the dairy industry to recover whey proteins from milk released as a result of the cheese-making process [103]. Membrane technology refers to the use of a semi-permeable membrane to separate a liquid into two different fractions by selectively allowing some compounds to pass through while impeding other compounds, typically based on molecular weight. Membrane technologies are promising alternative methods of enriching algal proteins, as well as developing novel techno-functional and bioactive ingredients. They have the advantage of being non-thermal and environmentally-friendly [103]. The most commonly used membrane technologies include microfiltration, ultrafiltration, nanofiltration, and reverse osmosis.

Membrane technologies could act as an alternative method for enriching algal proteins when used in conjunction with a cell disruption technique, such as polysaccharidase hydrolysis, UAE, or PEF. Disruption of the tough cell wall is a critical step required in order to increase the availability of algal proteins for extraction [82]. Membrane technologies are well suited for use with seaweed as part of a cascading biorefinery process to maximise valorisation of all components within algae, while avoiding the presence of heavy metals in the final product [104]. A combination of membrane technologies could be used to isolate algal proteins using the same principles of molecular weight cut-offs used in the dairy industry. In the dairy industry, microfiltration (MF) is used to extend the shelf-life of milk without any thermal treatment by removing microorganisms, while preserving overall taste and sensory attributes [105]. MF could be used to remove algae cell wall components and bacteria with a molecular weight greater than 200 kDa. Ultrafiltration (UF) could then be used to isolate proteins and other macromolecules between 1 and 200 kDa, similar to the way it is used in the dairy industry to generate enriched fractions less than 10, 5, 3 and 1 kDa. Nanofiltration (NF) could then be used to remove monovalent salts to minimise osmotic pressure, followed by reverse osmosis (RO) to reduce fluid volume [103].

Indeed, membrane technologies have already been used to isolate whole microalgae cells and several seaweed components. Tangential flow microfiltration was reported as an efficient method for recovering 70%–89% of algal biomass from wastewater treatments [106]. UF was previously used in conjunction with supercritical CO_2_ extraction and ultrasound to isolate *Sargassum pallidum* polysaccharides [107]. Polysaccharides with antioxidant activities were isolated from *U. fasciata* utilising hot water extraction followed by several stages of ultrafiltration with increasingly smaller pore sizes [108]. Furthermore, UF was used to isolate phycoerythrin protein from *Grateloupia turuturu* following cell homogenisation, which was reported to retain 100% of the protein without denaturation [109]. Alternatively, a two-stage ultrafiltration could be applied for algal protein enrichment, as demonstrated by the separation of polysaccharide components in *Tetraselmis suecica* using different sized pore membranes following high-pressure homogenisation [110].

## 4. Applications

### 4.1. Human Nutrition

Protein is an essential nutritional component in the diet of athletes, required to repair and build muscle tissue broken down during exercise, with the American College of Sports and Medicine recommending between 1.2 and 1.7 g protein per kg body weight [111]. Seaweed and microalgae are rich sources of protein and contain all of the essential amino acids at various concentrations [112]. Algae could therefore represent a valuable resource for athletes requiring high levels of protein, especially for vegan athletes for whom eggs and dairy whey protein may not be suitable [113].

Seaweed and microalgae have been used as a source of human nutrition for thousands of years in some indigenous populations [114]. One of the main reasons for the high consumption of seaweed and microalgae is due to their significant protein content, which is comparable to, or even greater than, some plant sources [28]. Some species of red seaweeds (Rhodophyta), such as *P. palmata* and *P. tenera*, have been reported to contain as much as 33% and 47% dw, respectively [3]. Similarly, some species of microalgae have been reported to contain even higher levels, as high as 63% dw in *Spirulina* sp. [115]. Microalgae are typically consumed as a dietary supplement in the form of powder, pills, or tablets [9]. However, they have also been incorporated into a number of functional foods, including noodles, bread, biscuits, drinks, sweets, and beer [116]. Several businesses have been set up for the sale of algal products, such as AlgaVia^®^ (www.algavia.com), which produces protein- and lipid-rich algal flour from *Chlorella protothecoides*.

There are several species of seaweed that have traditionally been consumed, largely due to their high protein content. For example, *P. tenera* (nori) is commonly used as a sushi wrap in several Asian cultures [22]. Many species of seaweed are particularly high in the amino acids aspartic acid and glutamic acid, which exhibit a unique and interesting flavour that led to the discovery of the taste sensation referred to as ‘umami’ [40]. The flavour enhancer monosodium glutamate was first discovered in the brown seaweed *L. japonica* (kombu), which has been found to particularly appeal to the umami taste sensation [117].

Although the consumption of seaweed in humans is currently underdeveloped, especially in Western countries, the high protein content and favourable essential amino acid profile makes seaweed a promising source of protein that is ripe for future expansion [40]. Seaweed has been successfully incorporated as a functional ingredient into several foods at the laboratory scale. *U. pinnatifida* (wakame) integrated into pasta has antioxidant activity and acceptable sensory attributes at levels up to 10% [118]. Bread containing 4% *A. nodosum* can significantly reduce energy intake in overweight individuals in the meal following enriched bread consumption [119]. Bread incorporating similar concentrations of renin-inhibitory peptides from *P. palmata* hydrolysates also had acceptable sensory attributes, with the bioactive properties reported as having survived the baking process [120].

*Spirulina* is the most highly consumed microalgae due to its high protein content and added nutritional benefits, including anti-hypertension, renal protective, anti-hyperlipidaemia, and anti-hyperglycaemic [121]. As well as being a rich source of proteins, *Spirulina* contains high levels of hypocholesterolemic γ-linoleic acid (GLA), B-vitamins, and free-radical scavenging phycobiliproteins [122]. It has therefore been given the label of a ‘super food’ by the World Health Organisation (WHO) and has even been sent to space by the National Aeronautics and Space Administration (NASA) due to its nutrient-dense properties [123]. As a demonstration of this, *Spirulina* has 180% more calcium than milk, 670% more protein than tofu, 3100% more β-carotene than carrots, and 5100% more iron than spinach [27]. The world’s largest producer of *Spirulina* is Hainan Simai Enterprising Ltd., which is located in the Hainan province of China [121]. This cultivation farm produces an annual 200 tonnes dried biomass, accounting for 25% of the national output and a considerable 10% of the global production. Alternatively, the Earthrise Company has the largest *Spirulina* production plant, which is located in California, USA, and covers 440,000 m^2^ [121].

*Chlorella* is another widely consumed microalga, with global sales exceeding US $38 billion [124]. The largest producer of *Chlorella* is Taiwan Chlorella Manufacturing and Co. (Taipei, Taiwan), which produces 400 tons dried biomass annually [121]. The main substance found in *Chlorella* that is beneficial for human health is β-1,3-glucan, which is an active immunostimulant, free-radical scavenger and reducer of blood lipids [125]. *Chlorella* is also rich in proteins (48% dw), polyunsaturated fatty acids (PUFAs) (39% of total lipids), and phosphorus (1761.5 mg/100g dw) [115].

### 4.2. Industrial Applications

Lectins and phycobiliproteins are two families of bioactive algal proteins which have been exploited for several industrial applications. Lectins are most commonly extracted from macroalgal sources, while phycobiliproteins are typically isolated from microalgae [126]. Phycobiliproteins, especially phycoerythrin, can constitute a significant proportion of the overall protein content in red algae, with levels of 1.2% (total dw) reported for *P. palmata* [127]. Lectins are found at similar levels and a yield of 1% lectins was obtained previously from *Eucheuma serra* (Rhodophyta) [128].

#### 4.2.1. Lectins

Lectins are glycoproteins known for their aggregation and high specificity binding with carbohydrates without initiating modification through associated enzymatic activity [129]. Lectins are involved in several biological processes, including host-pathogen interactions, cell–cell communication, induction of apoptosis, cancer metastasis and antiviral activities [130]. Due to their carbohydrate binding capacity with high specificity, lectins are used in blood grouping, anti-viral (including human immunodeficiency virus type 1(HIV-1)), cancer biomarkers, and targets for drug delivery [131]. Lectins derived from algae have not received the same level of characterisation compared to other plant-derived lectins. Nevertheless, some of the bioactivities that have been observed in algal lectins include mitogenic, cytotoxic, antibacterial, anti-nociceptive, anti-inflammatory, anti-viral (HIV-1), platelet aggregation inhibition, and anti-adhesion [132].

#### 4.2.2. Phycobiliproteins

Phycobiliproteins are water-soluble proteins with an important role in photosynthesis within cyanobacteria, Rhodophyta, and cryptomonads [133]. Phycobiliproteins are components of phycobilisomes, which are large light energy-capturing complexes anchored to thylakoid membranes [134]. There are four main divisions of phycobiliproteins which are grouped based on their colour and absorption characteristics, namely, phycoerythrin, phycocyanin, allophycocyanin, and phycoerythrocyanin [135]. The main commercial producers are *Spirulina* sp. (cyanobacterium) and *Porphydrium* sp. (Rhodophyta macroalgae) [121].

Phycobiliproteins are used in fluorescent labelling, flow cytometry, fluorescent microscopy, and fluorescent immunohistochemistry [136,137]. However, the primary commercial application of these phycobiliproteins appears to be as natural dyes, with phycocyanin in particular used as a blue pigment used in products such as chewing gum, popsicles, confectionary, soft drinks, dairy products, and wasabi, as well as cosmetic products, such as lipstick and eyeliner [121]. Several patents concerning health beneficial bioactivities of phycobiliproteins have also already been filed for nutraceutical applications such as anti-oxidative, anti-inflammatory, anti-viral, anti-tumour, neuroprotective, and hepatoprotective activities [135].

### 4.3. Animal Feed

The high protein content of algae can also be beneficial for use as animal feed, including aquaculture, farm animals, and pets. An estimated 30% of global algal production is estimated to be used for animal feed, with 50% of *Spirulina* biomass in particular used as feed supplement due to its excellent nutritional profile [124,138]. Several species of microalgae including *Spirulina*, *Chlorella*, and *Schizochytrium* sp., and seaweed including *Laminaria* sp. and *Ulva* sp. can be incorporated as protein sources into the diets of poultry, pigs, cattle, sheep, and rabbits [4,139]. Most of the research on the incorporation of algae as animal feed has been carried out with poultry, likely due to their promising prospects for improved commerciality [138].

Tasco^®^ is an example of a proprietary seaweed meal derived from *A. nodosum*, produced by Acadian Seaplants in Nova Scotia, Canada (http://www.acadianseaplants.com), which has demonstrated beneficial properties when included in animal feed [140]. Tasco^®^ has four main identified benefits for animal production, including resistance to stressors, improved immune system, increased productivity/quality, and a reduction in pathogenic microorganisms in the final meat product [141,142,143,144]. These benefits have been observed in several species, including monogastric and ruminant species, when at feed inclusion levels of 2% on a daily basis [145].

#### 4.3.1. Poultry

Supplementing poultry feed with microalgae as a protein source can improve their health, productivity, and value. This has been demonstrated using a variety of species, including *Chlorella* sp., *Arthrospira* sp., *Porphyridium* sp., and *Haematococcus* sp. [86,139,146,147]. Chickens fed with supplemented *Spirulina* have been reported to have increased viability, improved overall health and reduced plasma concentrations of cholesterol, triglycerides, and fatty acids [148]. These birds also appeared to have an improved immune system as demonstrated by a significant increase in white blood cell count and enhanced macrophage phagocytic activity [148,149]. Ross and Dominy (1990) reported that feeding White Leghorn cockerel chicks, Hubbard by Hubbard male broiler chicks, and Japanese quail with varying concentrations of *Spirulina* in dietary feed slightly delayed growth rates, but did not affect final growth at concentrations less than 10% [150]. Furthermore, this study also reported that the inclusion of *Spirulina* also increased fertility rates, as well as increasing the intensity of the egg-yolk colour [150]. These results have been confirmed by several other studies, indicating that the inclusion of *Spirulina* at a concentration of 2%–2.5% in the feed intensifies the colour of egg yolks to make it more esthetically pleasing for consumers [151,152]. The intensified colouration of the yolk is thought to be due to β-carotene [153]. The inclusion of *Spirulina* can also further valorise egg products by decreasing their cholesterol and saturated fatty acid content, and replacing it with increased levels of beneficial omega-3 polyunsaturated fatty acids [146,154].

Incorporation of 3% *U. lactuca* in broiler chickens increased breast muscle yield compared to birds solely fed corn diet, as well as decreased serum lipids, cholesterol, and uric acid concentrations [155]. The red seaweed *Polysiphonia* spp. was also demonstrated to improve pellet binding in duck feed at concentrations of 3%, as well as improve its overall nutrient profile [156]. The incorporation of red seaweeds *C. crispus* and *Sarcodiotheca gaudichaudii* was also reported to effectively act as a prebiotic to improve chicken gut health, productivity, and egg quality [157].

#### 4.3.2. Pigs

The replacement of up to 33% of soy proteins with proteins from *Arthrospira maxima*, *A. platensis*, and *C. vulgaris* in pig feed has been reported as being suitable without any adverse effects [158]. The effect of feed processing appears to play a role in the utility of *Spirulina* in pig’s feed. The addition of *Spirulina* to pellets was reported to decrease average daily gain, whereas incorporation of *Spirulina* to meal diets actually increased average daily gain [159]. Addition of *Spirulina* to the diet has also been suggested to improve fertility in pigs, increasing sperm motility and storage viability [160].

Supplementation of the brown seaweed *L. digitata* increased pig body weight gain by 10%, as well as increase the concentration of iodine in fresh muscle by 45%, thus increasing its valorisation [161]. Similarly, *A. nodosum* has also been reported to increase iodine content in pig tissue, while also increasing the concentrations of beneficial bacteria within the gut [162]. However, these results are in contrast to the findings of Reilly and colleagues (2008), who reported that the brown seaweeds *Laminaria hyperborea* and *L. digitata* actually decreased the biodiversity of beneficial microbial populations within the pig’s gut, although this did not significantly affect the pig’s performance [163].

#### 4.3.3. Ruminants

Of all the animals evaluated for algae supplementation, ruminants are the most promising in terms of digesting the high fibre content for the greatest extraction efficiency of algal proteins [139]. This is in contrast to mono-gastric animals, for which it has been suggested that some form of prior processing may be required in order for animals (and humans) to utilise algal proteins more efficiently [164]. Cattle will preferentially drink water containing 20% suspended *Spirulina*, increasing their daily water intake by 24.8 g/kg [165]. Furthermore, this study also reported that 20% of the consumed *Spirulina* bypasses degradation within the rumen, allowing for increased digestion and absorption of protein and nutrients within the abomasum [165]. Incorporation of 200 g/day *Spirulina* with cattle feed was reported to be an economically effective method of increasing animal body weight (8.5%–11%) and daily milk production (21%) [166]. As well as increasing milk quantity, *Spirulina* supplementation has also been demonstrated to increase milk quality by decreasing saturated fatty acids, while simultaneously increasing monounsaturated fatty acids and polyunsaturated fatty acids [167]. Similar results were observed in other studies, as well as with supplementation of *Schizochytrium* sp. [168,169].

Sheep have also been demonstrated to benefit from microalgae as a protein source, with lambs reported to have increased average daily gains upon consumption of 10 g of *Spirulina* per day [170]. Similarly, *Spirulina* diet supplementation increased the feed intake of rabbits, as well as improve the quality of rabbit meat with higher levels of GLA [171]. The green seaweed *U. lactuca* has been reported as a suitable low-energy, high-protein foodstuff for sheep and goats [172,173]. However, seaweed may be not be suitable for supplementation in pregnant ewes, having been reported to interfere with passive immunity in lambs and increasing mortality rate [174].

### 4.4. Aquaculture

Microalgae are vital for the artificial reproduction of several aquaculture species, especially molluscs [138]. Microalgae also play an important role in aquaculture, other than as a food source for zooplanktons by stabilising pH, reducing bacterial growth, and improving the quality of rearing medium [175]. This leads to improved survival and growth compared to that of clear water fed with artificial diets [176]. Microalgae are the natural base of the entire aquatic food chain. This has led to their widespread incorporation as an important food source and feed additive in the commercial rearing of many aquatic animals, including molluscs, shrimp, and rotifers [177]. Filtering molluscs are by far the greatest consumer of microalgae in aquaculture, with 10.1 × 10^6^ tonnes produced in 1999, compared to shrimp (1.2 × 10^6^ tonnes), and small larvae fish, such as sea breams and flatfish (177,400 tonnes) [178].

Replacements of live microalgae are already commercially available (such as *Chaetoceros* 1000 “Premium Fresh” Instant Algae™ paste, Liqualife™ liquid larval feed, Zeigler™ E-Z Larvae liquid feed, and Zeigler™ Z-Plus feed), but typically provide inferior growth and survival rates [179]. For example, the survival rate of brown larval shrimp (*Farfantepenaeus aztecus*) significantly decreased from 90.86 ± 3.19% when fed live microalgae, to 14.865 ± 14.35% in shrimp fed with 100% replacement E-Z larvae [179].

Microalgae are also often used as a dietary supplement to refine the products of aquaculture and increase their valorisation. Carotenoid pigments, such as astaxanthin derived from *Haematococcus pluvalis*, are incorporated into the diets of salmonoids, shrimp, lobsters, and crayfish, to give them their characteristic pink flesh [180]. Similarly, inclusion of astaxanthin at a concentration of 30 ppm significantly increases the colour pattern and intensity of ornamental fish, including tetras, cichlids, gouramis, damos, goldfish, and koi, increasing their market value several fold [147].

Red seaweed has been suggested as a promising protein source feed additive. Incorporating 10% *Gracilaria chilensis* in the diet of Atlantic salmon (*Salmo solar*) was reported to significantly increase specific growth rate by 1.51% ± 0.12% compared to the control diet [181]. Including 1.0% and 10% *G. chilensis* in the diets of *S. solar* was also suggested to increase antiviral activity against the infectious salmon anaemia (ISA) virus. Similarly, inclusion of 5% and 15% *P. palmata* was reported to improve hepatic function and have a positive effect on body lipid content in *S. salar* compared to the control diets [182]. Wild abalone are opportunistic feeders that consume a variety of macroalgae species and typically have increased growth rate in captivity when fed a diet with several species compared to single species diets [183]. *Haliotis tuberculata coccinea* fed with a mixed diet including *Ulva rigida*, *Hypnea spinella*, and *Gracilaria cornea* displayed significantly greater growth rates, length, and weight gain than diets consisting of single algal species [184]. Seaweed is also often used in the feed of sea cucumber culture systems, which are used for human consumption in many Asian countries. *L. japonica* and *U. lactuca* have been reported as an economical additive to the diets of sea cucumber *Apostichopus japonicas* with low ammonia–nitrogen production and suitable digestibility [185].

### 4.5. Bioactive Peptides

Bioactive peptides are particular amino acid sequences that can have additional physiological health benefits beyond their basic nutritional value [126]. These peptides are typically between 2 and 30 amino acids in length and have hormone-like properties. Bioactive peptides are inactive within the parent proteins, but can be released through fermentation or hydrolysis. Milk proteins remain the most common source for bioactive peptides [186,187]. However, bioactive peptides have also been identified in a number of food sources, including meat, egg, fish, and blood, as well as plant sources, including rice, soybean, wheat, pea, broccoli, garlic, and algae [132,188,189,190,191,192,193,194,195,196,197].

Bioactive peptides have been found to have a multitude of beneficial effects, including anti-hypertensive, anti-oxidative, antithrombotic, hypocholesterolemic, opioid, mineral binding, appetite suppression, anti-microbial, immunomodulatory, and cytomodulatory properties [187]. Bioactive peptides from algae have also been found to display bioactive properties, although they are not as well characterised compared to peptides from other sources (Table 2) [198]. Antimicrobial peptides have been identified from *Saccharina longicruris* protein hydrolysates, significantly decreasing the growth rate of *Staphylococcus aureus* [199]. The hexapeptide Glu-Asp-Arg-Leu-Lys-Pro isolated from *Ulva* sp. was demonstrated to have mitogenic activity in skin fibroblasts [200].

Owing to the high levels of oxidative stresses and free radicals in their environment, microalgae and seaweed have developed many defensive systems that can stimulate antioxidant activity when consumed [213]. Antioxidant peptides have therefore been isolated from several species of microalgae, including *C. vulgaris*, *Navicula incerta*, and *Chlorella ellipsoidea* [214,215,216,217]. Antioxidant peptides have also been isolated from various Irish and Korean brown seaweeds, which indicated that those with higher phenolic content, such as *Ecklonia cava* and *Sargassum coreanum*, correlated with increased antioxidant activity [218,219,220]. Peptides displaying antioxidant and anticancer bioactivity have also been reported in Sri Lankan red and green seaweed, of which *Caulerpa racemosa* demonstrated the most promising free radical scavenging and anticancer bioactivity [221].

Microalgae have also been reported to display anticancer peptides, such as *Chlorella pyrenoidosa* antitumor polypeptide (CPAP) derived from *Chlorella pyrenoidosa* and polypeptide Y2 derived from *A. platensis* [222,223]. Protein hydrolysates from *Porphyra columbina* phycocolloid extraction by-products were reported to have immunosuppressive, anti-hypertensive, and antioxidant capacities [224]. Anti-inflammatory peptides have been isolated from microalgae, such as *Chlorella* 11-peptide derived from *C. pyrenoidosa* [225], as well as peptides derived from *A. maxima* (Leu-Asp-Ala-Val-Asn-Arg and Met-Met-Leu-Asn-Phe), which were additionally reported to display anti-atherosclerosis bioactivity [226,227]. Similarly, anti-atherosclerosis peptides have also been isolated from *P. palmata* and were shown to be non-toxic in Zebrafish at a concentration of 1 mg/mL [228].

Algal peptides have been reported to display several other bioactivities, including hepatoprotective, immunomodulatory, ultraviolet (UV) radiation-protective, anti-osteoporosis, and anti-coagulant [229,230,231,232,233,234]. Finally, it is important to note that several studies have reported that short algae-derived peptides are capable of resisting gastrointestinal digestion from enzymes such as trypsin, pepsin, and chymotrypsin [203,207,235]. This is an essential trait for bioactive peptides in order to achieve their physiological effect at their site of action [236].

#### Anti-Hypertensive Peptides

**Figure 2:**
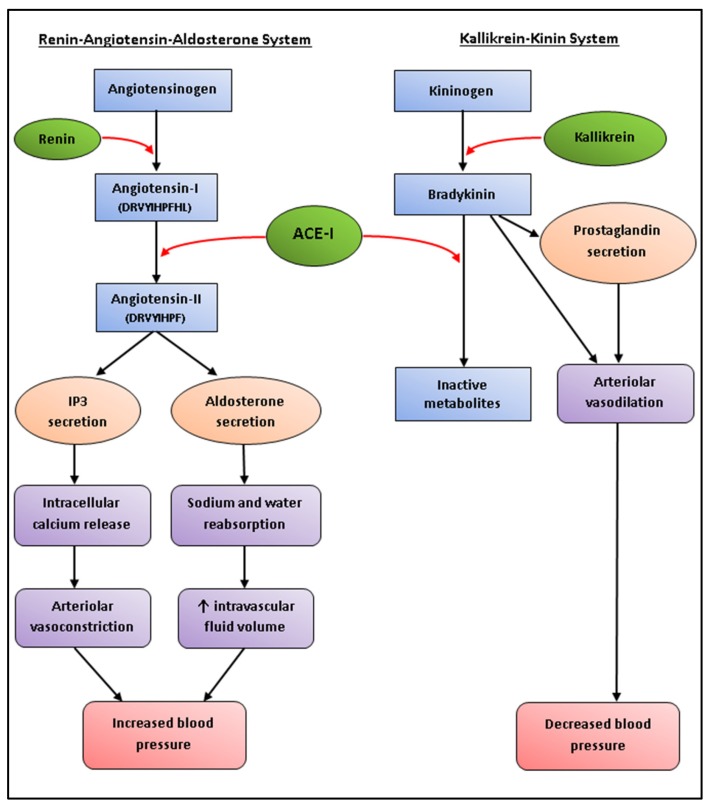
Schematic representation of the renin–angiotensin–aldosterone system (RAAS) and the hypertensive effect of angiotensin-I-converting enzyme (ACE-I). Angiotensinogen is converted to the decapeptide angiotensin-I by renin. ACE-I cleaves the C-terminal dipeptide His-Leu of angiotensin-I to form angiotensin-II. Binding of angiotensin-II to its receptor (AT1) stimulates the secretion of inositol 1,4,5-triphosphate (IP3) and aldosterone, which induce arteriolar vasoconstriction and increased intravascular fluid volume, respectively, resulting in increased blood pressure. Within the kallikrein–kinin system, kallikrein converts kininogen to bradykinin, which induces arteriolar vasodilation by prostaglandin secretion and binding of bradykinin with its receptor, resulting in decreased blood pressure. However, the hypotensive effect of bradykinin is largely dependent on the rate of degradation by ACE-I, which hydrolyzes bradykinin to form inactive metabolites.

Hypertension is the single largest risk factor attributed to deaths worldwide, making it an ideal target for bioactive peptides [237]. Angiotensin-I-converting enzyme (ACE-I) is a proteolytic enzyme that affects vasoconstriction in two major blood pressure regulatory systems, namely renin-angiotensin–aldosterone system (RAAS) and kinin–kallikrein system, leading on to the development of hypertension (Figure 2) [126]. ACE-I inhibitors have therefore become one of the most commonly studied targets, and with global annual sales exceeding US $6 billion, ACE-I inhibitory drugs can be considered as one of the major protease inhibitor success stories [238]. Synthetic ACE-I inhibitor drugs, such as captopril, enalapril, and alacepril, often come with several side effects, including hypotension, dry cough, and impaired renal function [19]. Function foods with anti-hypertensive bioactivities have therefore become a popular alternative to synthetic drugs, especially for individuals who are borderline hypertensive and do not warrant the prescription of pharmaceutical drugs [239].

ACE-I-inhibitory peptides have been isolated from a variety of seaweed and microalgae sources [132]. Four tetrapeptides were identified from peptic digests of *U. pinnatifida* which displayed in vitro ACE-I inhibitory and in vivo anti-hypertensive bioactivity in spontaneously hypertensive rats (SHRs) (Table 2) [201]. Suetsuna and colleagues also identified several dipeptides from *U. pinnatifida* using hot water extraction [202]. Both single administration and repeated dose administration of four of the dipeptides significantly reduced blood pressure in SHRs. For example, a single administration of 50 mg/kg body weight Tyr-His, Phe-Tyr, and Ile-Tyr lowered systolic blood pressure (SBP) by 50, 46, and 33 mm Hg, respectively, after 3 h [202]. The dipeptide Lys-Tyr took slightly longer to have the greatest effect, with a reduction in SBP of 45 mm Hg observed after 6 h. Similarly, one week of continuous oral administration of 10 mg/kg body weight/day Tyr-His, Lys-Tyr, Phe-Tyr, and Ile-Tyr reduced SBP by 34, 26, 34 and 25 mm Hg, respectively [202]. Similarly, Sato and colleagues (2002) also identified ACE-I inhibitory peptides from *U. pinnatifida* which displayed in vitro and in vivo bioactivity [203].

Dipeptides were isolated from wakame Protease S “Amano” hydrolysates by three-step high-performance liquid chromatography. The peptides showed strong ACE-I inhibitory potency and four of the dipeptides (Val-Tyr, Ile-Tyr, Phe-Tyr, and Ile-Trp) were reported to significantly reduce blood pressure in SHRs comparable to that of the positive control (captopril) when administered at a dose of 1 mg/kg body weight (Table 2) [203]. Cha and colleagues (2006) reported promising peptides derived from digestion of 70 °C aqueous extracts of *E. cava* using five proteases from Novo Co. (Novozyme Nordisk, Bagsvaerd, Denmark), including alcalase, flavourzyme, kojizyme, neutrase, and protamex [204]. ACE-I inhibitory peptides have also been isolated from *P. columbina*, *P. palmata*, *Bangia fusco-purpurea*, *P. yezoensis*, and *H. fusiformis* (Table 2) [205,206,207,235,240].

In addition to seaweed, ACE-I inhibitory bioactive peptides have also been isolated from a number of microalgae species. Suetsuna and Chen (2001) identified several peptides from *C. vulgaris* and *A. platensis* which displayed promising ACE-I inhibitory and anti-hypertensive activity in SHRs (Table 2) [208]. *Nannochloropsis oculata* has been shown to be a promising source of commercially exploitable biodiesel due to its high lipid content (up to 28.7% dw) [209]. In order to maximise its potential, while minimising waste disposal costs, biodiesel by-products were analysed for ACE-I inhibitory peptides. The commercial enzymes Alcalase, Neutrase, Flavourzyme, Pancreatic Trypsin Novo (PTN), and Protamex were used to generate hydrolysates, of which the Alcalase-generated peptide Leu-Val-Thr-Val-Met was identified as the most potent [209]. In a similar study, *N. oculata* was also digested with a variety of proteases, including pepsin, trypsin, α-chymotrypsin, papain, Alcalase, and Neutrase for the generation of ACE-I inhibitory hydrolysates [210]. The pepsin hydrolysates exhibited the highest ACE-I inhibitory bioactivity, of which two peptide sequences (Gly-Met-Asn-Asn-Leu-Thr-Pro and Leu-Glu-Gln) in particular were identified as having the greatest activity (Table 2). Hydrolysis of *C. ellipsoidea* using the proteases Protamax, Kojizyme, Neutrase, Flavourzyme, Alcalase, trypsin, α-chemotrypsin, pepsin, and papain generated several ACE-I inhibitory peptides, of which Val-Glu-Gly-Tyr was shown to be the most potent in vitro ACE-I inhibitor and in vivo anti-hypertensive with SHRs (Table 2) [211].

An anti-hypertensive peptide (Ile-Arg-Leu-Ile-Ile-Val-Leu-Met-Pro-Ile-Leu-Met-Ala) has also been isolated from *P. palmata*, which was reported to inhibit renin at concentrations similar to that that of the positive control [241]. Renin catalyzes the initial rate-limiting step within the RAAS, and is therefore an important target for the treatment of high blood pressure (Figure 2) [242].

## 5. Challenges

### 5.1. Access Rights

At present, all seaweed species are harvested by hand in Ireland. This includes the most economically valuable species brown seaweed *Ascophyllum nodosum* and the red calcified coralline seaweeds *Phymatolithon purpureum* and *Lithothamnion corallioides* (commonly called maërl) [243]. The legislation for harvesting seaweed in Ireland is based on the Foreshore Acts 1933–1998, which states that the Department of Communications Marine and Natural Resources is empowered to grant licences for seaweed harvesting on the seabed out to 12 nautical miles [244]. There are currently no restrictions on harvesting quantities in Ireland, nor are there any restrictions on harvesting times. The only exception to this is for maërl due to its extremely slow growth rate (0.6–1.5 mm per annum) [245]. However, the future implementation of mechanical harvesting methods will likely require a review of existing legislation to ensure appropriate access rights and sustainable harvesting yields are maintained. Mechanical harvesting of *Laminaria* spp. is already present in various European countries, such as France and Norway, providing a valuable resource for making any such revisions [243].

### 5.2. Variability

An additional challenge, which is particularly relevant in the production of bioactive peptides, are the high levels of variability in algal proteins. The protein content can vary by season, temperature, and location in which the seaweed is harvested [46]. The relative composition of particular proteins within the plant can also differ, changing the concentrations of amino acids and therefore altering the yield of desired peptides as a consequence [246]. For example, annual monitoring of *P. palmata* harvested on the French Atlantic coast showed that protein levels were highest in the winter and spring months, varying from 9 to 25%, and peaking in May [247]. Similarly, *Gracilaria cervicornis* and *S. vulgare* varied by season, with protein levels negatively correlating with temperature and salinity [248]. Different harvest locations of *U. pinnatifida* in New Zealand also significantly affected the protein content and amino acid composition [249].

### 5.3. Scalability

The scalability of protein extraction from algae is a further obstacle that needs to be overcome before seaweed and microalgae become a viable source. Algal protein extraction is still very much in its infancy, meaning that many of the methods that have been developed are still at small scale [132]. PEF and ultrasound have been suggested as being suitable for large scale algal protein extraction [250]. Membrane technologies may also be scalable for commercial applications, with ultrafiltration reported as being suitable for R-phycoerythrin extraction from the seaweed *G. turuturu* at the industrial scale [109].

### 5.4. Digestibility

One of the most important challenges for extracting proteins from algae is the high levels of cell wall anionic polysaccharides which can become bound to the proteins and create viscous medium, further increasing the difficulty of protein extraction [46]. The morphology of different seaweed species has been suggested to be an important factor in determining protein yield, with tougher thallus forms reported to require increased processing [68]. Several brown algae species (*Eisena bicyclis*, *H. fusiformis*, *U. pinnatifida*) were reported to have reduced in vitro digestibility (51.8%–57.1%) compared to red algae *P. palmata* and *P. tenera* (70.2% and 87.3%, respectively), which was suggested to have been due to increased levels of dietary fibre [34]. Furthermore, the type of polysaccharide has been shown to influence the level of protein digestion. *P. palmata* and *G. verrucosa*, both red seaweeds, were shown to have significantly different levels of in vitro corrected nitrogen digestibility (1.9% and 16.3% after 6 h, respectively) [251]. This was largely attributed to differences in polysaccharide fractions (pentoses and hexoses, respectively).

Treatments to disrupt the cellulosic cell wall could help to overcome this issue to make algal proteins and other cell components more accessible [28]. Heat treatment is one such option that can improve food taste, texture, safety in terms of allergenicity and microbial load, and preservation, while also increasing bioavailability and utilisation of proteins by partial denaturation and breakdown of proteins into peptides, allowing for easier access by proteolytic enzymes [252]. An example of this can be seen with boiling of *P. palmata* , resulting in a 64%–96% increase in liberated amino acids [253]. Alternatively, fermentation can also increase protein digestibility due to the degradation of insoluble fibres, such as xylan. This can be seen with the fermentation of *P. palmata * using the fungal mould *Trichoderma pseudokoningii* which was found to decrease the xylan content from 53% to 19% dw [75]. Washing in distilled water has also been shown to be a simple, yet effective method for improving in vitro digestibility by solubilising and removing mineral salts [75].

Alternatively, the method of drying seaweed has been shown to influence algal protein digestibility. Compared to freeze-drying, oven-drying was demonstrated to significantly improve (*p* < 0.05, two-way ANOVA) the protein extractability and in vitro bioaccessibility of brown seaweed species *Sargassum hemiphyllum*, *Sargassum henslowianum*, and *Sargassum patens* [45]. The increased extractability from oven-drying were suggested to have been due to the decomposition of phenolic compounds at the high temperatures, as well as increased disruption of anionic or neutral polysaccharides found within the cell wall of the seaweed [45].

Protein digestibility can also be largely influenced by the presence of phenolic compounds present in algae, especially phlorotannins in brown seaweeds [45]. Oxidised phenolic compounds can react with amino acids to form insoluble complexes, which may inhibit proteolytic enzymes and thus decrease their nutritive values [65]. The negative correlation between phenolic content and digestibility was demonstrated by *U. lactuca* which was reported to have higher phenolic content (38.8 ± 0.5%) compared to the red algae *H. charoides* (16.9% ± 1.0%) and *H. japonica* (16.3% ± 0.03%) and thus, lower bioaccessibility (85.7% ± 1.9%, 88.7% ± 0.7%, and 88.9% ± 1.4%, respectively) [65]. This is likely to be a greater issue in brown algae, as these are typically higher in phenolics, including catechins, flavanols, and phlorotannins [10,67].

### 5.5. Food Safety

While algae can be natural accumulators of vitamins and minerals, they can also gather toxic elements, such as heavy metals. There are therefore strict legal limits in Europe for the safe maximum exposure of heavy metals, such as mercury, arsenic, lead, and cadmium, in foods for human consumption. These limits are based upon the recommendations of the Joint FAO/WHO Expert Committee on Food Additives (JECFA) and the Panel on Contaminants in the Food Chain of the European Food Safety Authority (CONTAM Panel), and are outlined in the legislation under Commission Regulation (EC) No. 1881/2006 [254].

The tolerable weekly intake (TWI) of methylmercury, arsenic, lead, and cadmium are 1.6, 15, 25, and 25 μg/kg body weight, respectively [255,256,257,258]. The CONTAM panel has reported that the current lead TWI (15 μg/kg body weight) may no longer be appropriate due to low levels found in foodstuffs, while arsenic TWI (1.6 μg/kg body weight) should be lowered due to cancers and adverse effects observed at current legal limits [256,257]. Lead and mercury appear to occur in algae at levels that are safe for human consumption [259,260]. In contrast, arsenic and cadmium have been identified in algae at levels above the legal limits, with *H. fusiformis* in particular containing a high content of these toxic heavy metals [259,260]. Microalgae do not appear to exceed legal levels of heavy metals [261].

The lack of regulation for algae as a dietary supplement is of additional concern, leading to large variations between different batches from the same producer [262]. This has led to concerns being raised about quality and contamination at the production and processing stages [263]. Downstream applications for which the algal proteins are intended can play a role in determining processing methods. For example, food grade reagents and processes may be required for proteins intended for human consumption, whereas this may not be such an issue with proteins intended for commercial applications or animal feed [264]. Furthermore, any reagents used for the production or extraction of algal proteins must also be environmentally friendly with minimum impact on local ecosystems [265]. Following protein extraction, greater emphasis must additionally be placed on protein purification, with salt removal and buffer exchange being important steps [266]. Membrane technologies, such as ultrafiltration and nanofiltration, have already been implemented in this context within the dairy industry, although these technologies could also be used for other food applications, including algal proteins [267].

### 5.6. Price

A critical factor that will determine the commercial viability of microalgae and seaweed is their competitiveness compared to other sources on the market. For example, a major application for the production of microalgae is the extraction of lipids to be converted to biofuel. However, while they are a greener alternative for the environment, they are still not competitive compared to fossil-based petroleum fuels [266]. Algae competitiveness could be further increased by taking a holistic view and maximising the extraction of all available high-value components by cascading biorefinery. Conventional methods of algal protein extraction are often quite wasteful as they typically dispose of the algae by-products following processing [88]. Seaweed is ideally suited for cascading biorefinery because it contains many high-value components as well as bulky low-value components that are considered raw materials for the bio-based economy, such as xylose and glucose [268]. As several studies have shown, the by-products of algae can still have many applications that could be of economic value [209,215,224]. Further research is needed to develop new methods that will enable algal protein production, extraction and processing costs to be lowered.

## 6. Discussions

Seaweed and microalgae are generally considered to be a promising source of nutrition, rich in vitamins, minerals, and protein. Some species have even been reported to display protein at concentrations greater than traditional plant and animal protein sources [28]. Within these protein sequences, bioactive peptides have been identified with beneficial properties for human health, including antioxidant, anticancer, anti-hypertensive, immunosuppressive, anti-atherosclerosis, and hepatoprotective effects [198]. Algae have also been reported to contain high quality proteins in terms of EAA content [3], as well as displaying in vitro protein digestibility that is comparable to commonly consumed plants [67]. However, there are conflicting reports about the bioavailability of algal proteins when examined in vivo. Several studies have demonstrated that consumption of raw, unprocessed seaweed can result in reduced growth [43,44]. In contrast, other studies have reported opposite findings, with seaweed proteins suggested as being a beneficial dietary supplement for improved animal growth, meat quality, and valorisation [155,161,162]. Furthermore, microalgae have long been used as the basal diet in the rearing of animals in aquaculture, especially molluscs [138].

One of the major reasons for reduced protein digestibility was suggested to be due to the high fibre content that makes up the algal cell wall [46]. Algae typically contain high levels of polysaccharides, although this can vary significantly in seaweed (4%–76% dw) and microalgae (8%–64% dw) according to species and time of harvest [22,28]. Similarly, phenolic compounds can react with amino acids to form insoluble compounds [65]. Great emphasis has therefore been placed on researching methods for breaking down of the anionic cell wall in order to allow the release of the valuable intracellular contents [13].

Conventional protein extraction methods, such as enzymatic hydrolysis and physical processes, are laborious, time-consuming, and may require the use of solvents [13]. Novel protein extraction methods, such as UAE, PEF, and MAE, have thus been investigated as a means of overcoming these limitations. UAE is a cost-effective and low-solvent consumption method for extraction of high-value compounds. UAE is already widely used at commercial scale in food processing and could therefore also be implemented for the extraction of algal proteins [81]. PEF for the extraction of intracellular algal components is still in its infancy, having predominantly been used for lipid extraction [93]. Nevertheless, its lack of solvent consumption or heat gives it potential for the future extraction of algal proteins [100]. MAE has been poorly studied in algae, although the high temperature involved would likely make it unsuitable for extraction of proteins [81]. Alternatively, membrane filtration technologies, such as those used in the dairy industry, could also be considered a gentle method for protein enrichment when used in conjunction with cell disruption methods. MF has already been demonstrated for harvesting whole microalgal cells, while UF was reported for the extraction of algal polysaccharides [106,107]. However, there remains to be a distinct lack of studies investigating the use of membrane technologies in the enrichment of algal proteins.

In conclusion, algae have been reported as a valuable source of nutrition. However, there are inconclusive reports about the digestibility of algae and the bioavailability of the proteins within. More research is needed to investigate the bioavailability of algal proteins in vivo. Cell disruption techniques will likely play an essential role in the successful extraction and enrichment of algal protein ingredients at a commercial scale. The most promising methods that have been used in algae to date are UAE and PEF, both of which are non-thermal, require low solvent consumption, and have been implemented at commercial scale for the extraction of compounds in other food processes. Alternatively, membrane technologies are a method for isolating compounds which show great promise, but have yet to be investigated sufficiently in algae.

## Figures and Tables

**Table 1:** Conventional pre-treatment cell disruption methods and extraction methods for precipitating proteins from seaweed. Dry weight; dw.

Extraction Method	Species	Extraction Name	Reagents	Protein Yield	Reference
Enzymatic hydrolysis	*Palmaria palmata*	Polysaccharidase degradation	Cellulase (Cellucast^®^) and xylanase (Shearzyme^®^)	Factor 3.3 compared to control	[46]
	*Chondrus crispus*, *Gracilaria verrucosa*, and *Palmaria palmata*	Polysaccharidase degradation	κ-carrageenase, β-agarase, xylanase, cellulase	-	[74]
	*Palmaria palmata*	Polysaccharidase degradation	Cellulase (Cellucast^®^), xylanase (Shearzyme^®^) and Ultraflo^®^ (β-glucanase)	11.57 ± 0.08 g/100 g dw (67% yield)	[73]
Physical Process	*Porphyra acanthophora* var. *acanthophora*, *Sargassum vulgare* and *Ulva fasciata*	Aqueous treatment and Potter homogenisation	Ultra-pure water	8.9 g/100 g dw, 6.9 g /100 g dw, 7.3 g /100 g dw	[68]
	*Palmaria palmata*	Osmotic stress	-	6.77 ± 0.22 g/100 g dw (39% yield)	[73]
		High shear force	-	6.92 ± 0.12 g/100 g dw (40% yield)	
Chemical extraction	*Ascophyylum nodosum*	Acid-alkaline treatment	0.4 M HCl and 0.4 M NaOH	59.76% yield	[69]
	*Ulva rigida*	Two-phase system	NaOH and 2-mercaptoethanol	-	[70]
	*Ulva rotunda*				
	*Laminaria digitata*	Two-phase system	Polyethylene glycol (PEG) and potassium carbonate	-	[71]
	*Palmaria palmata*	Alkaline and aqueous	NaOH and N-acetyl- l-cysteine (NAC)	4.16 g/100 g dw (24% yield)	[73]

**Table 2:** Angiotensin-I-converting enzyme (ACE)-I inhibitory bioactive peptides derived from seaweed and microalgae. The potency of the peptides is indicated by their IC_50_ values, which refers to the concentration required to inhibit enzyme activity by 50%.

Source	Hydrolytic Method	Peptide Sequence	IC_50_	Reference
*Undaria pinnatifida* (wakame)	Pepsin	Ala-Ile-Tyr-Lys	213 μM	[201]
		Tyr-Lys-Tyr-Tyr	64.2 μM	
		Lys-Phe-Tyr-Gly	90.5 μM	
		Tyr-Asn-Lys-Leu	90.5 μM	
*Undaria pinnatifida* (wakame)	Hot water extraction	Tyr-His	5.1 μM	[202]
		Lys-Trp	10.8 μM	
		Lys-Tyr	7.7 μM	
		Lys-Phe	28.3 μM	
		Phe-Tyr	3.7 μM	
		Val-Trp	10.8 μM	
		Val-Phe	43.7 μM	
		Ile-Tyr	2.7 μM	
		Ile-Trp	12.4 μM	
		Val-Tyr	11.3 μM	
*Undaria pinnatifida* (wakame)	Protease S “Amano”	Val-Tyr	35.2 μM	[203]
		Ile-Tyr	6.1 μM	
		Ala-Trp	18.8 μM	
		Phe-Tyr	42.3 μM	
		Val-Trp	3.3 μM	
		Ile-Trp	1.5 μM	
		Leu-Trp	23.6 μM	
*Ecklonia cava*	Alcalase	Enzymatic digest	2.79 μg/mL	[204]
	Flavourzyme	Enzymatic digest	3.56 μg/mL	
	Kojizyme	Enzymatic digest	2.33 μg/mL	
	Neutrase	Enzymatic digest	3.10 μg/mL	
	Protamex	Enzymatic digest	3.28 μg/mL	
*Porphyra yezoensis*		Ile-Tyr	2.69 μM	[205]
		Met-Lys-Tyr	7.26 μM	
		Ala-Lys-Tyr-Ser-Tyr	1.52 μM	
		Leu-Arg-Tyr	5.06 μM	
*Hizikia fusiformis*		Gly-Lys-Tyr	3.92 μM	[206]
		Ser-Val-Tyr	8.12 μM	
		Ser-Lys-Thr-Tyr	11.07 μM	
*Palmaria palmata (dulse)*	Thermolysin	Val-Tyr-Arg-Thr	0.14 μM	[207]
		Leu-Asp-Tyr	6.1 μM	
		Leu-Arg-Tyr	0.044 μM	
		Phe-Glu-Gln-Trp-Ala-Ser	2.8 μM	
*Chlorella vulgaris*	Pepsin	Ile-Val-Val-Glu	315.3 μM	[208]
		Ala-Phe-Leu	63.8 μM	
		Phe-Ala-Leu	26.3 μM	
		Ala-Glu-Leu	57.1 μM	
		Val-Val-Pro-Pro-Ala	79.5 μM	
*Arthrospira platensis*		Ile-Ala-Glu	34.7 μM	
		Phe-Ala-Leu	11.4 μM	
		Ala-Glu-Leu	11.4 μM	
		Ile-Ala-Pro-Gly	11.4 μM	
		Val-Ala-Phe	35.8 μM	
*Nannochloropsis oculata*	Alcalase	Leu-Val-Thr-Val-Met	18.0 μM	[209]
*Nannochloropsis oculata*	Pepsin	Gly-Met-Asn-Asn-Leu-Thr-Pro	123 μM	[210]
		Leu-Glu-Gln	173 μM	
*Chlorella ellipsoidea*	Protamex, Kojizyme, Neutrase, Flavourzyme, Alcalase, trypsin, α-chymotrypsin, pepsin, and papain	Val-Glu-Gly-Tyr	128.4 μM	[211]
*Chlorella vulgaris*	Flavourzyme, alcalase, papain, and pepsin	Val-Glu-Cys-Tyr-Gly-Pro-Asn-Arg-Pro-Gln-Phe	29.6 μM	[212]

## Abbreviations

EAA(s)	essential amino acid(s)
FAO	Food and Agriculture Organisation of the United Nations
TNOASR	The Netherland Organisation for applied scientific research
TIM	TNOASR’s Intestinal Model
UAE	ultrasound-assisted extraction
PEF	pulsed electric field
MAE	microwave-assisted extraction
MF	microfiltration
UF	ultrafiltration
WHO	World Health Organisation
ACE-I	angiotensin-I-converting enzyme
RAAS	renin-angiotensin-aldosterone system
SHR(s)	spontaneously hypertensive rat(s)
SBP	systolic blood pressure
